# Neutrophil extracellular traps in urinary tract infection

**DOI:** 10.3389/fped.2023.1154139

**Published:** 2023-03-20

**Authors:** Katarína Krivošíková, Nadja Šupčíková, Alexandra Gaál Kovalčíková, Jakub Janko, Michal Pastorek, Peter Celec, Ľudmila Podracká, Ľubomíra Tóthová

**Affiliations:** ^1^ Department of Pediatrics, National Institute of Children’s Diseases and Faculty of Medicine, Comenius University, Bratislava, Slovakia; ^2^ Institute of Molecular Biomedicine, Faculty of Medicine, Comenius University, Bratislava, Slovakia; ^3^ Institute of Pathophysiology, Faculty of Medicine, Comenius University, Bratislava, Slovakia

**Keywords:** uropathogenic bacteria, innate immune response, pyelonephritis, cystitis, NETosis

## Abstract

**Background:**

Urinary tract infections (UTI) are common types of bacterial infection in children. UTI treatment is aimed to prevent complications including hypertension, proteinuria, and progression to chronic kidney disease. Activated neutrophils release chromatin-based structures associated with antimicrobial proteins called neutrophil extracellular traps (NETs). We aimed to describe the role of NET-associated markers in children with UTI as well as the role of NETs formation in a mouse model of UTI.

**Materials and methods:**

Markers of NETs including extracellular DNA (ecDNA), myeloperoxidase (MPO) and cathelicidin were analyzed in children with febrile UTI caused by *E.* *coli* (*n* = 98, aged 0.3–1.3 years) and in healthy controls (*n* = 50, 0.5–5.2 years). Moreover, an acute experimental model of UTI was performed on PAD4 knock-out mice with diminished NETs formation (*n* = 18), and on wild-type mice (*n* = 15).

**Results:**

Children with UTI had significantly higher urinary NETs markers including total ecDNA, nuclear DNA and mitochondrial DNA, altogether with MPO and cathelicidin. The concentrations of MPO and cathelicidin positively correlated with ecDNA (*r* = 0.53, *p* ≤ 0.001; *r* = 0.56, *p* ≤ 0.001, respectively) and the number of leukocytes in the urine (*r* = 0.29, *p* ≤ 0.05; *r* = 0.27, *p* ≤ 0.05, respectively). Moreover, urinary MPO was positively associated with cathelicidin (*r* = 0.61, *p* ≤ 0.001). In the experimental model, bacterial load in the bladder (20-fold) and kidneys (300-fold) was significantly higher in PAD4 knock-out mice than in wild-type mice.

**Conclusion:**

Higher urinary NETs makers—ecDNA, MPO and cathelicidin and their correlation with leukocyturia in children with UTI confirmed our hypothesis about the association between NETs and UTI in children. Higher bacterial load in mice with diminished NETs formation suggests that NETs are not only a simple consequence of UTI, but might play a direct role in the prevention of pyelonephritis and other UTI complications.

## Introduction

Urinary tract infections (UTI) are the most common type of bacterial infection in the pediatric population (1, 2). During early infection neutrophils, as the first line of the innate immune response towards pathogen, employ several strategies, such as degranulation, cytokine production, phagocytosis, or release fibrous DNA-histone complexes, called neutrophil extracellular traps (NETs) (3, 4). NETs are coated with proteins and antimicrobial peptides of both nuclear and granular origin (3). Granular components of NETs together with DNA are generally considered markers of NETs. This is of special importance since there is no gold standard method that would reliably assess NETs production (5), especially in the urine.

Apart from antimicrobial defense of neutrophils (6, 7), extracellular DNA (ecDNA) released during NETs formation is recognized as a damage-associated molecular pattern that further activates immune response. According to recent studies, markers of NETs formation can be perceived as markers of inflammation in a variety of pathologies including sepsis, multiple trauma, obesity, systemic autoimmune diseases and thrombosis (8–15). Recently, studies of Yu and colleagues identified proteins derived from neutrophils (16) and NETs-like structures (17) in urine sediment of UTI patients. However, the role of NETs formation and cleavage in the pathogenesis of UTI is understudied.

Therefore, we have aimed to investigate the role of NETs formation and inflammation in UTI. Firstly, we investigated the presence of urinary markers of NETs in children with UTI in order to clarify the associations between UTI severity and NETs formation. Secondly, the role of NETs in the pathogenesis of UTI was further elucidated in an animal UTI model using protein-arginine deiminase type 4 (PAD4) deficient mice. PAD4 enzyme is crucial for chromatin depolymerization during NETs formation (18, 19). We hypothesized that children with UTI have increased concentrations of NETs markers released from activated neutrophils in urine that play role in the inhibition of bacteria spreading, and thus PAD4 knock-out mice, with a limited ability to form NETs, will have higher bacteria invasion of bladder and kidneys.

## Materials and methods

### Clinical part

In this cross-sectional study, 179 consecutive pediatric patients with acute pyelonephritis were enrolled. Patients were admitted to the Department of Pediatrics of the National Institute of Childreńs Diseases in Bratislava, Slovakia between March 2019 and March 2022. Diagnosis of pyelonephritis was based on the medical history and physical examination, a urine microscopy and urine culture. Patients had to meet all of the following criteria: presence of nonspecific symptoms (fever above 38°C, irritability, poor feeding), C-reactive protein above 50 mg/L, pyuria and significant bacteriuria. Pyuria was defined as positive leukocyte esterase (LE > trace) on dipstick analysis and/or >10 white blood cells (WBC) per one power field in centrifuged urine. Significant bacteriuria was defined as >50,000 CFU/ml of a single uropathogen from a catheterized specimen (20). Only children with significant bacteriuria caused by *E. coli* were enrolled to this study.

Overall, 179 patients were sampled, however 81 patients were excluded from the study due to pyuria without significant bacteriuria (38 patients), significant growth of other uropathogens than *E. coli* (40 patients), and the co-existence of other acute or chronic disorders (3 patients). Ninety-eight patients (median age: 0.8, IQR: 0.3–1.3 years) with acute pyelonephritis caused by *E. coli* strain were included for further analysis (Table 1). None of the patients had been diagnosed with recurrent UTI.

**Table 1:** Clinical and demographic data of pediatric patients with UTI.

Parameter	UTI	CTRL
Age (years)	0.8 (0.3–1.3)	2.4 (0.5–5.2)
Sex (F/M)	67/31	24/26
WBC (10^9^/L)	19.8 (15.1–23.7)	ND
ANC (10^9^/L)	10.6 (6.3–14.2)	ND
ALC (10^9^/L)	5.7 (4.3–7.5)	ND
AMC (10^9^/L)	2.3 (1.6–2.9)	ND
AEC (10^9^/L)	0.08 (0.03–0.2)	ND
ABC (10^9^/L)	0.05 (0.04–0.07)	ND
RBC (10^12^/L)	4.1 (3.7–4.3)	ND
PLT (10^9^/L)	412 (328–538)	ND
Creatinine (µmol/L)	21 (18–26)	ND
BUN (mmol/L)	2.7 (2.0–3.2)	ND
UA (µmol/L)	192 (166–240)	ND
TP (g/L)	62.4 (58.4–67)	ND
Albumin (g/L)	38.6 (36.3–41.6)	ND
CRP (mg/L)	78.5 (59.9–157.5)	ND
Bilirubin (µmol/L)	7.6 (4.1–13.2)	ND
Natrium (mmol/L)	136 (135–138)	ND
Potassium (mmol/L)	4.7 (4.4–5.0)	ND
Calcium (mmol/L)	2.54 (2.46–2.62)	ND
Phosphorus (mmol/L)	1.60 (1.34–1.78)	ND
Chloride (mmol/L)	102 (100–104)	ND
Magnesium (mmol/L)	0.94 (0.88–0.98)	ND

ABC, absolute basophil count; AEC, absolute eosinophil count; ALC, absolute lymphocyte count; AMC, absolute monocyte count; ANC, absolute neutrophil count; BUN, blood urea nitrogen; CRP, C-reactive protein; PLT, platelets count; ND, not determined; RBC, red blood cells; TP, total proteins; UA, uric acid; WBC, white blood cells. Results are expressed as median (interquartile range) (skewed data).

A control group of 50 healthy children was recruited among children attending regular check-ups during visits at general pediatrician or from the children attending outpatient visits at the Department of Pediatrics due to disorders unrelated to UTI (short height, deformities, or hemangioma; median age: 2.4, IQR: 0.5–5.2 years). None of the healthy controls had a history of UTI.

#### Sample collection

All blood and urine samples were collected at the time of admission, i.e., before initiation of antibiotics treatment. Venous blood was collected from either dorsal superficial vein or cubital vein into K_3_EDTA, lithium-heparin and SST™ *ΙΙ* Advance tubes (BD Vacutainer Plastic K_3_EDTA Tube, Becton Dickinson, Heidelberg, Germany). Urine samples were collected into sterile Falcon tubes (Sarstedt, Nümbrecht, Germany) *via* urinary catheterization or *via* sterile bag specimen (healthy children during regular visits). Standard biochemical analysis and urine microscopy were performed immediately. If urine cannot be cultured within 4 h of collection, the sample was refrigerated. Aliquots of blood and urine were centrifuged at 1,600 g for 10 min. The supernatants were stored at −20°C until further analysis.

#### Biochemical analysis

Complete blood count with differential leukocyte count was assessed using a Sysmex XN-1000™ Hematology Analyzer (Sysmex Group’s, Kobe, Japan). Serum creatinine, BUN, uric acid, total proteins, albumin, bilirubin, C-reactive protein, natrium, potassium, calcium and phosphorus were analyzed using standard laboratory methods (Cobas c501 analyzer, Roche Diagnostic, Basel, Switzerland).

Urinary creatinine was measured using Jaffé reaction as described previously (21). Briefly, 10 microliters of samples were mixed with 200 microliters of freshly prepared reagent consisted of 0.2 M sodium hydroxide and 25 mM picric acid (5:1 ratio). Absorbance was measured at 492 nm. Absorbance measured after 1st minute was subtracted from the absorbance measured at 6th minute.

Concentration of myeloperoxidase (MPO) in urine was determined using commercially available spectrophotometric assay according to manufactureŕs protocol (Human Myeloperoxidase DuoSet Elisa Kit, R&D Systems, Minneapolis, MN, United States). Absorbance was measured at 450 nm. Limit of detection was 62.5 pg/ml.

Similarly, cathelicidin in urine was measured by commercial Elisa kit as recommended by the manufactureŕs protocol (LL-37 Human Elisa kit, Hycult Biotech, Wayne, PA, United States). Urine samples were 10-times diluted. Absorbance was measure at 450 nm. Limit of detection was 0.14 ng/ml.

#### Isolation and quantification of ecDNA

To remove apoptotic bodies, aliquots of plasma and urine used for the ecDNA isolation were centrifuged second time at 16,000 g for 10 min. ecDNA was isolated from 200 microliter of samples based on column method using commercial kit (QIAamp DNA Mini Kit, Qiagen, Hilden, Germany) as recommended by manufactureŕs protocol. Total ecDNA was assessed *via* fluorometric method using Qubit Fluorometer and Qubit dsDNA HS Assay Kit (Invitrogen, Carlsbad, CA, United States). Cellular origin of ecDNA was determined using quantitative polymerase chain reaction recorded in real time. Real-time PCR was performed using SsoAdvanced Universal SYBR Green Supermix (Bio-Rad, Hercules, CA, United States) on MasterCycler RealPlex (Eppendorf, Hamburg, Germany). To quantify ncDNA, primers encoding human beta-globin gene (F: 5’-GCT TCT GAC ACA ACT GTG TTC-3’, R: 5’-CAC CAA CTT CAT CCA CGT TCA-3’) with following program: initiation 3 min at 98°C, 40 × 15 s at 98°C for denaturation, 30 s at 51°C for annealing, 30 s at 60°C for polymerization, were used. To determine mtDNA, primers were designed to target mitochondrial D-loop amplification (F: 5’-CAT AAA AAC CCA ATC CAC ATC A-3’, R: 5’-GAG GGG TGG CTT TGG AGT-3’) using following program: initiation 3 min at 98°C, 40 × 15 s at 98°C for denaturation, 30 s at 47°C for annealing, 30 s at 60°C for polymerization. Concentrations of ncDNA and mtDNA in urine are expressed in genomic equivalent per milligram of creatinine in urine (GE/mg of creatinine).

### Animal experiment

All procedures were performed on 7–8 weeks old mice obtained from Animalab (Prague, Czech Republic) strains C57BL/6 (*n* = 15) and PAD4^−/−^ KO (*n* = 18). Mice were housed in cages with a controlled temperature and humidity and 12/12 h light/dark cycle. All animals had free access to standard chow and water during the whole experiment.

#### Bacterial strain and growth condition

Uropathogenic *E. coli* strain CFT073 isolated from patient with acute pyelonephritis (22). Bacteria were grown statically at 37°C in liquid Luria-Bertani medium for ∼16 h, centrifuged at 7,500 g for 10 min at 4°C. The pellet was resuspended in ice-cold sterile phosphate buffer saline to final concentration of bacteria OD_600nm _= 1–8 × 10^8^ colony-forming units (CFU)/ml.

#### Experimental design

For induction of UTI in mice, surgical approach of bladder inoculation was used (23). Briefly, animals were anesthetized with isoflurane and small vertical incision in prepubic region was made to expose the bladder. The bladder was directly inoculated with 50 µl of ∼4 × 10^7^ CFU. After 24 h the animals were sacrificed by cervical dislocation and bladders and kidneys were harvested. Organs were immediately homogenized in sterile ice-cold phosphate buffered saline and serial dilutions of the homogenates were plated on agar plates. The plates were incubated overnight at 37°C, bacterial colonies were counted, and the number of CFU per organ were calculated.

### Statistical analysis

GraphPad Prism software 9.4.1 was used for statistical analysis (GraphPad Software, San Diego, California, United States). Normality of data was tested using D'Agostino-Pearson omnibus test. Outliers were identified using Grubbś test. Differences of non-normally distributed variables between two independent groups were evaluated by Mann-Whitney *U* test was used. Data with high variability were log transformed. Associations between variables and clinical parameters were determined using Spearman’s test (skewed data). *p* value less than 0.05 was considered as a limit of statistical significance. Data are presented as median with interquartile range.

### Ethical approval

All procedures performed in this study involving animals were conducted in accordance with the ethical standards of the institutional and national research committee and was approved by the Ethics Committee of the Institute of Pathophysiology, Faculty of Medicine, Comenius University, Bratislava, Slovakia and the State Veterinary and Food Administration of the Slovak Republic. Clinical part of the study was performed according to guidelines of the Declaration of Helsinki and was approved by the Ethics Committee of the National Institute of Children’s Diseases at the Faculty of Medicine, Comenius University, Bratislava, Slovakia. Consent to participate of all children was provided by a parent or legal guardian.

## Results

### Clinical study

#### Concentrations of ecDNA, MPO and cathelicidin

**Figure 1:**
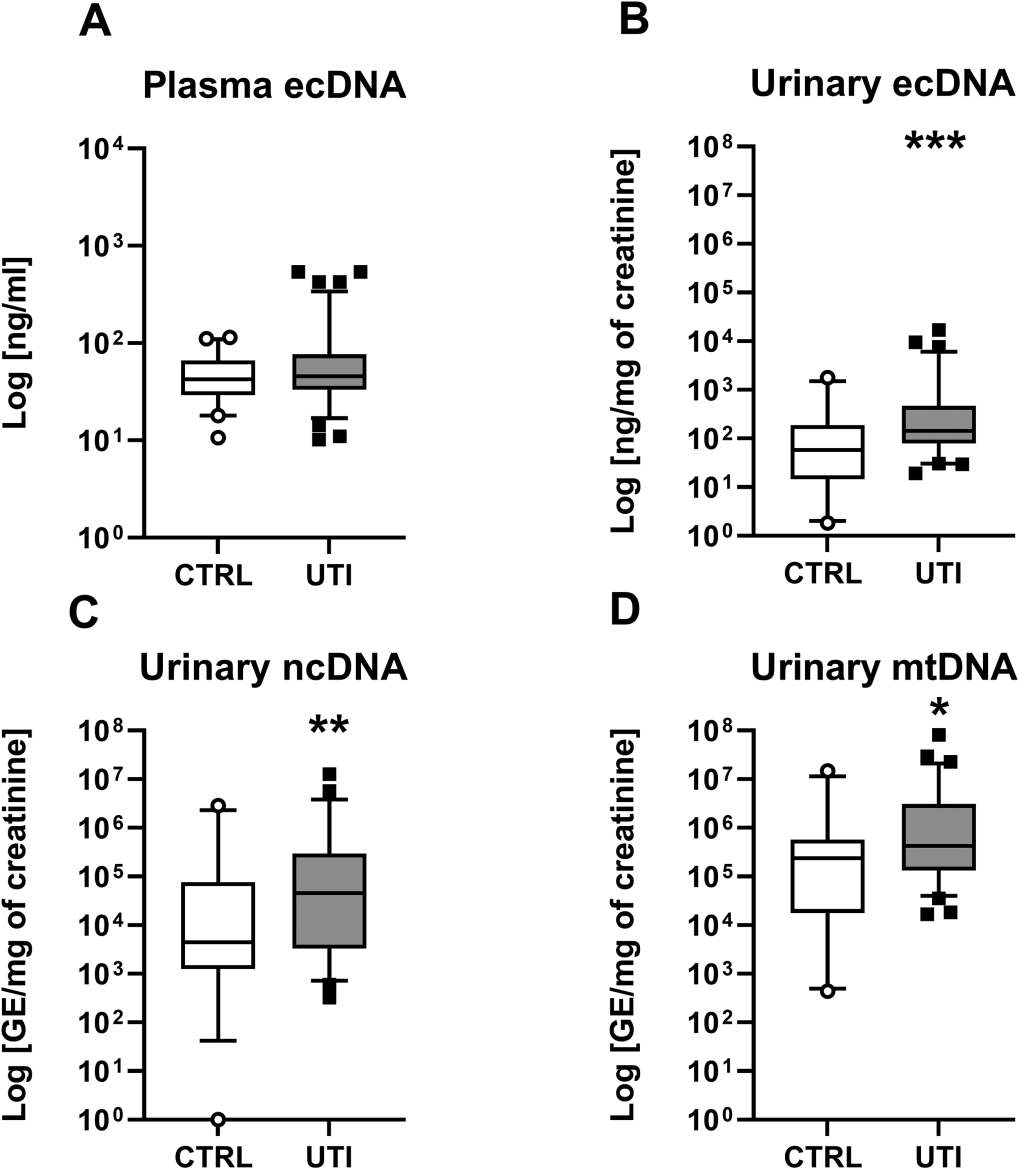
Concentration of extracellular DNA (ecDNA) in children with urinary tract infection (UTI) and in healthy controls (CTRL). (**A**) Plasma total ecDNA, (**B**) Urinary total ecDNA, (**C**) Urinary nuclear DNA (ncDNA), (**D**) Urinary mitochondrial DNA (mtDNA). Results are presented using box plots with median and interquartile range. Data were compared using Mann–Whitney *U* Test. *Denotes *p* < 0.05, **denotes *p *< 0.01, ***denotes *p *< 0.001 in comparison to healthy children.

**Figure 2:**
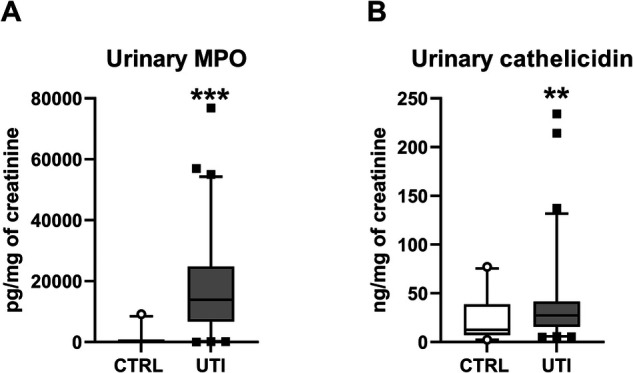
Concentration of antimicrobial peptides in urine of children with urinary tract infection (UTI) and in healthy controls (CTRL). (**A**) Myeloperoxidase (MPO), (**B**) Cathelicidin. Results are presented using box plots with median and interquartile range. Data were compared using Mann–Whitney *U* Test. **Denotes *p *< 0.01, ***denotes *p *< 0.001 in comparison to healthy children.

Children with UTI had similar concentration of total ecDNA in plasma as healthy children (Mann-Whitney *U* = 1,924, *z* score = −0.86, *p* > 0.05, Figure 1A). Concentrations of total ecDNA in urine were significantly higher in patients with UTI when compared to healthy controls (Mann-Whitney *U* = 556, *z* score = −3.12, *p* < 0.01, Figure 1B). Additionally, UTI patients displayed significantly higher concentrations of urinary ncDNA than healthy controls (Mann-Whitney *U* = 488, *z* score = −2.48, *p* < 0.05, Figure 1C). Correspondingly, urinary mtDNA in UTI patients was significantly higher in comparison to controls (Mann-Whitney *U* = 638, *z* score = −2.36, *p* < 0.05, Figure 1D). Similarly, concentrations of urinary MPO in children with UTI were higher than in healthy controls (Mann-Whitney *U* = 85.5, *z* score = −6.82, *p* < 0.001, Figure 2A). Additionally, UTI patients had significantly higher concentrations of urinary cathelicidin in comparison to controls (Mann-Whitney *U* = 612, *z* score = −2.44, *p* < 0.01, Figure 2B).

#### Relation of ecDNA with inflammatory parameters and NETs markers

Total plasma ecDNA in UTI patients did not correlate either with WBC, ANC, or with CRP (*r* = 0.07, *p* = ns; *r* = 0.04, *p* = ns; *p* = −0.007, *p* = ns). Similarly, total ecDNA in urine did not correlate with inflammatory parameters (*r* = 0.16, *p* = ns; *r* = 0.07, *p* = ns; *p* = −0.06, *p* = ns).

**Figure 3:**
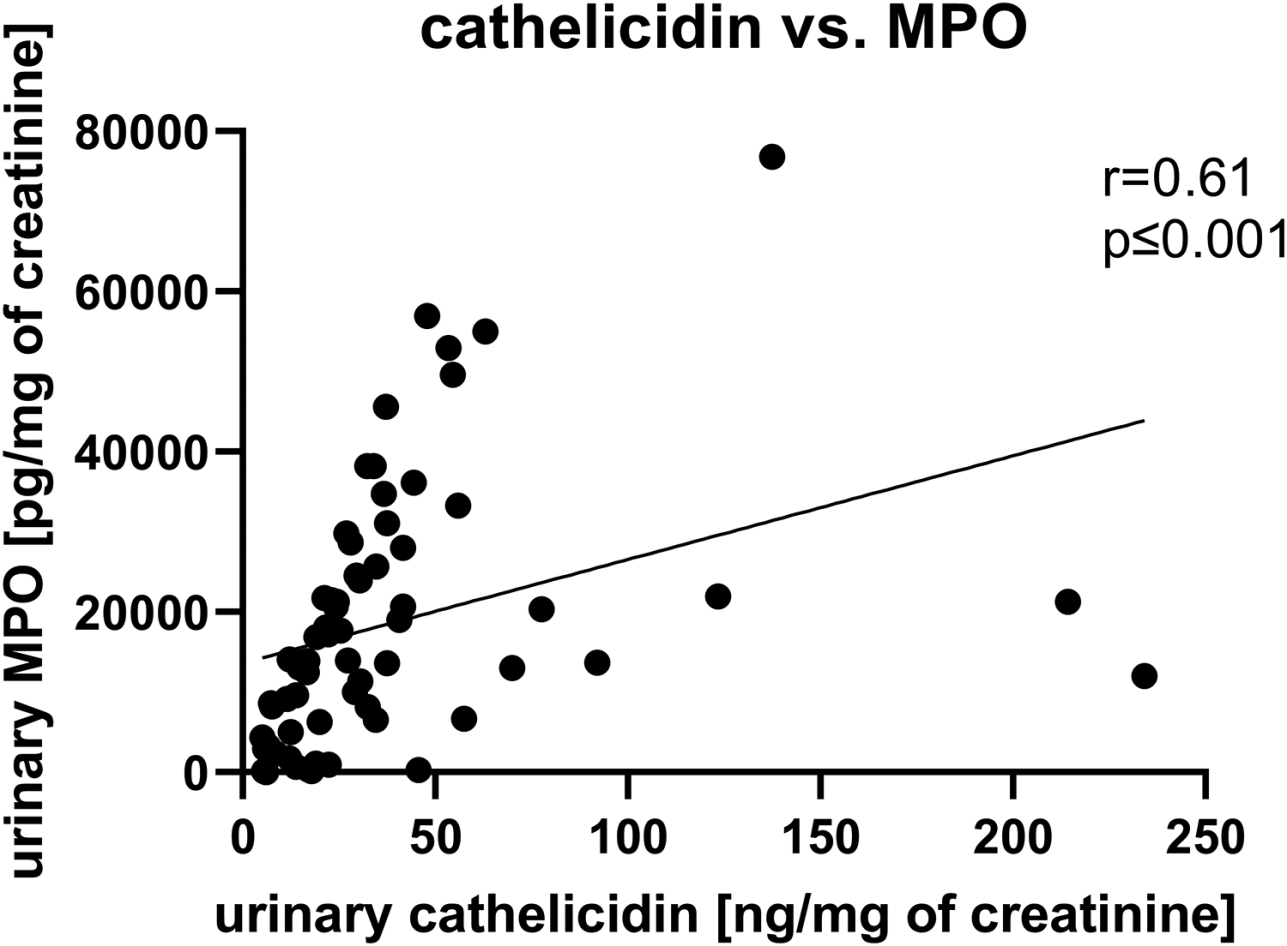
Correlation between cathelicidin and myeloperoxidase (MPO) in urine of children with urinary tract infection (UTI). Relations were tested using Spearman’s rank correlation test. *p* values less than 0.05 are considered as statistically significant.

In urine of UTI patients MPO positively correlated with concentrations of total ecDNA, ncDNA and mtDNA (Table 2). Further, Spearman’s correlation analysis revealed positive relation of cathelicidin with either total ecDNA, ncDNA or mtDNA (Table 2). Additionally, cathelicidin positively correlated with concentration of MPO in urine (*r* = 0.61, *p* < 0.001, Figure 3). Leukocyturia correlated with MPO and cathelicidin (Supplementary Material).

**Table 2:** Correlation between antimicrobial peptides and ecDNA in urine of children with UTI.

	ecDNA	ncDNA	mtDNA
Plasma MPO	*r* = 0.53	*r* = 0.46	*r* = 0.61
	*p* < 0.001	*p* < 0.001	*p* < 0.001
Urinary cathelicidin	*r* = 0.56	*r* = 0.58	*r* = 0.55
	*p* < 0.001	*p* < 0.001	*p* < 0.001

MPO, myeloperoxidase; ecDNA, extracellular DNA; ncDNA, nuclear DNA; mtDNA, mitochondrial DNA. Relations were tested using Spearman’s rank correlation test.

### Animal experiment

#### Validation of diminished NETs formation in PAD4^−/−^ KO mice

Polymorphonuclear cell isolated from WT and PAD4^−/−^ KO mice were incubated with phorbol 12-myristate 13-acetate (PMA, 500 nM) and uropathogenic *E. coli* (MOI 20) for 4 h. Colocalization of signal from DNA-binding dye SYTOX GreenTM (green channel) with strong signal from antibody against citrullinated histone H3 (red channel) was observed in PMN isolated from WT mice after incubation with PMA and *E. coli*, but not in PMN from PAD4^−/−^ KO mice (Supplementary Figure S2).

#### Determination of bacterial load

**Figure 4:**
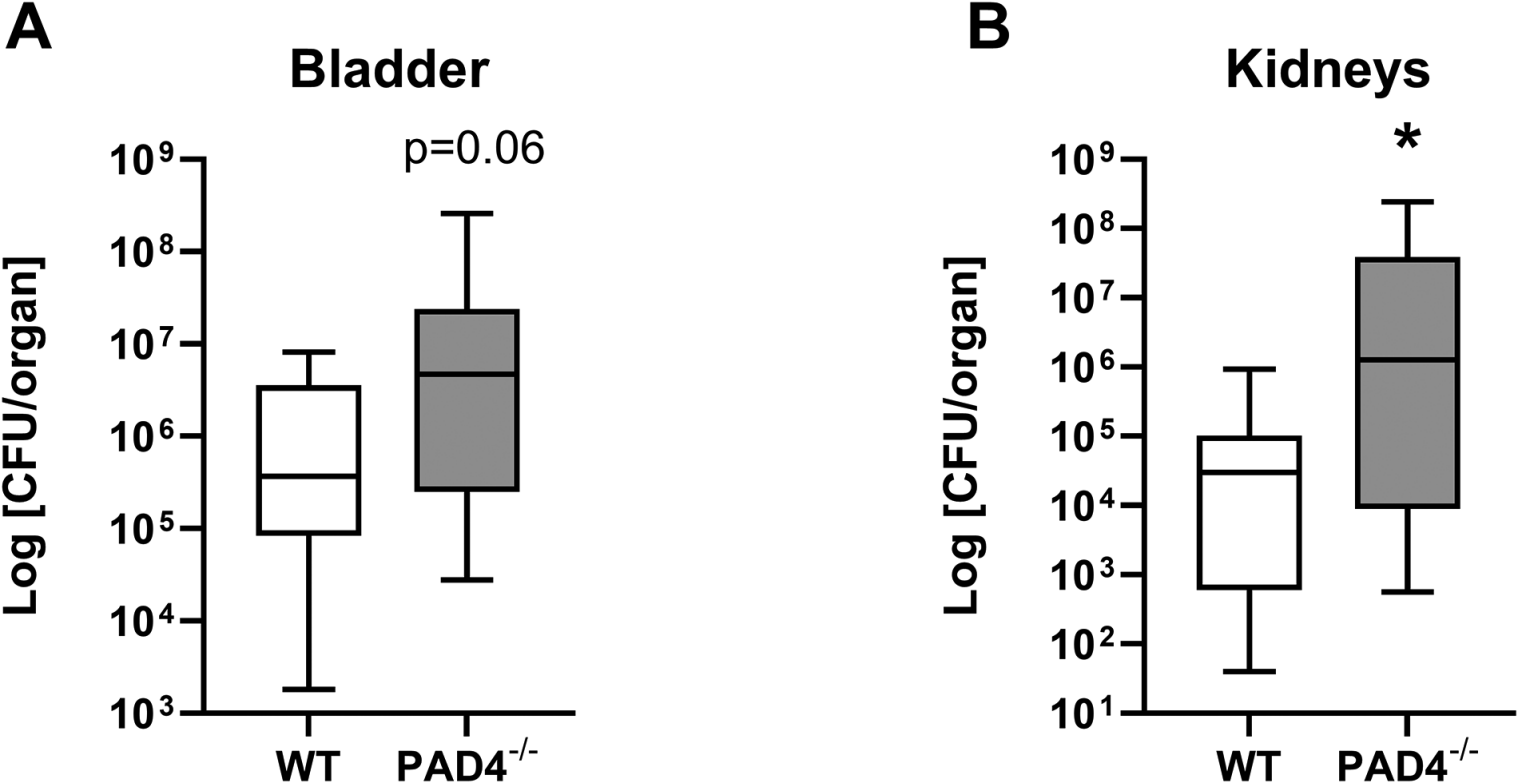
Concentrations of bacteria in urinary tract of mice infected by *E. coli*. Bacterial load in WT (*N* = 15) and PAD4^−/−^ (*N* = 18) mice. Mice were infected with uropathogenic *E. coli* (∼4 × 10^7^ CFU) and sacrificed 24 h post infection. Harvested organs were homogenized and plated using single plate-serial dilution spotting method. Colony forming units (CFU) were calculated in (**A**) bladder and (**B**) kidneys homogenates after overnight cultivation and compared with Mann–Whitney *U* Test. Results are presented using box plots with median and interquartile range.

Twenty-four hours post-infection, bacterial load in bladder in PAD4^−/−^ KO mice tended to be higher than in WT mice (Mann-Whitney *U* = 78, *z* score = −1.82, *p* = 0.06, Figure 4A). Correspondingly, PAD4^−/−^ KO mice had significantly higher concentration of bacteria present in kidneys compared to WT mice (Mann-Whitney *U* = 77, *z* score = −2.09, *p* < 0.05, Figure 4B).

## Discussion

The analysis of our data has shown that the concentration of total ecDNA in the urine rises with increasing inflammation. Plasma ecDNA concentrations did not change, which was in line with our hypothesis to focus on urine ecDNA as a suitable biomarker for the early detection and severity of acute pyelonephritis. Elevated levels of plasma ecDNA have been described in association with systemic infectious processes such as sepsis (24, 25). Urinary ecDNA of both nuclear and mitochondrial origin was elevated in our study. In similar work, Moreira et al. quantified ecDNA in urine of 55 adult patients with UTI reporting significantly higher concentrations when compared to 124 healthy individuals (26). To the best of our knowledge, no similar investigation has ever been conducted on children with UTI.

The rise of urine ecDNA was further accompanied by increased concentrations of MPO and cathelicidin. MPO as a well-established marker of inflammation and oxidative stress plays a central role in the microbial killing. Multiple studies display the presence of MPO in a variety of renal diseases (27–29). Ciragil et al. showed significantly elevated levels of MPO in patients with UTI than in healthy controls (30). They also determined high sensitivity (87%) and specificity (100%) values for MPO activity. Bai et al. demonstrated that urine MPO to creatinine ratio positively correlated with leukocytosis in patients with UTI and could even be used for initial inference of infectious bacterial types (31). Chromek et al. proved in their study of children with acute cystitis and pyelonephritis that while low concentrations of cathelicidin could be detected in sterile urine, these were significantly increased during inflammation (32). Considering our aim to investigate the relationship of NETs and inflammation in children with UTI, we have expected the rise of all discussed parameters, since ecDNA altogether with both antimicrobial granule components, MPO and cathelicidin, represent structural components of NETs. NETs formation is initially regulated as a reaction of neutrophils to microbial presence. However, with the increasing severity of the inflammatory reaction, damage to the own tissues stimulating further NETs production may occur at the same time (33, 34). This is supported by the positive correlations of total ecDNA together with ncDNA and mtDNA with MPO, cathelicidin as well as with leukocyturia in our study.

In our study, we confirmed using fluorescent microscopy that PAD4 KO mice do not form NETs *in vitro*. We also showed that PAD4 KO mice have higher bacterial load of the kidneys suggesting that NETs formation is not only a consequence of UTI, but it is directly involved in the immune protection against pathogens and prevention of UTI complications in our animal model. NETs seem to be an important component involved in the bacteria trapping in UTI. Our results are in line with previous study dealing with the role of NETs in pathogen-induced acute lung injury where PAD4 deficiency was associated with the increased bacterial load and inflammation in lungs (35). Similarly, PAD4 inhibition was associated with increased inflammation and bacteremia in a mouse model of necrotizing enterocolitis (36). On the contrary, other study of Claushuis et al. showed NET-like structures in lungs of both WT and PAD4 KO mice. Moreover, both groups revealed similar bacterial load, inflammation and organ injury (37). Correspondingly, PAD4 deficiency did not affect bacteremia in animal model of sepsis (38). Likewise, recent study argue against PAD4 unique role in NETs formation, and depending on the stimuli other PAD enzymes including PAD2 may be crucial for NETs formation, as well (39, 40). Therefore, further studies should evaluate the effect of different inhibitors of NETs formation as potential therapeutic targets. Although PAD4 deficiency in 24 h model of UTI was associated with a higher bacterial burden, this does not exclude NETs inhibition as a treatment approach. The used genetic model does not allow to test different timing of interference with NETs which might be of importance as shown for sepsis (41, 42).

The main limitation in the experimental study is that small volume of urine obtained from mice prevented evaluation of NETs, and thus only bacterial burden was reported. Limitation of the clinical part of the study is its cross-sectional design. Long-term observations would be valuable for gaining further insight. On the other hand, the clinical study was performed on a large cohort of pediatric patients that were strictly selected—all patients were aged under 2 years and were diagnosed with the first febrile UTI without known congenital abnormalities of kidneys or urinary tract. Moreover, patients with other uropathogen than *E. coli* were excluded from analyses. Children with UTI, instead of adults, represent a uniform cohort with bacterial inflammation of only one organ without any comorbidities that could affect concentration of NETs markers. Thus, unique design of the study minimized external factors of variability. Using NETs markers for an early diagnostic of UTI without the need to wait for the results from the urine culture might be of practical importance. PAD4 deficiency was associated with increased bacterial burden in kidneys, which suggests a protective role of NETs during acute phase of UTI, where they would halt the dissemination of bacteria. On the other hand, excessive NETs formation has been linked to tissue damage and exacerbation of pathological inflammation and further studies that will monitor NETs formation in a longer time-period are needed. Persistent NETs formation that might have potentially detrimental effects could thus be targeted by NETs formation inhibitors, further highlighting the importance of establishment of reliable urinary NETs markers.

## Data Availability

The raw data supporting the conclusions of this article will be made available by the authors, without undue reservation.
