# Supplementary material for: Neutrophil extracellular traps in urinary tract infection
Source: Front Pediatr. 2023 Mar 20;11:1154139. doi: 10.3389/fped.2023.1154139 (PMC10067609; doi:10.3389/fped.2023.1154139)
Supplement: Supplementary file 1 [file Datasheet1.pdf]

Supplementary Material

Neutrophil extracellular traps in urinary tract infection

Katarína Krivošíková[1†], Nadja Šupčíková[2†], Alexandra Gaál Kovalčíková[1, 2], Jakub Janko[2], Michal Pastorek[2], Peter Celec[2, 3], Ľudmila Podracká[1], Ľubomíra Tóthová[2*]

*** Correspondence:**

Assoc. prof. Ľubomíra Tóthová, Dr., PhD

Institute of Molecular Biomedicine

Faculty of Medicine, Comenius University

Sasinkova 4, 811 08 Bratislava

Slovakia

tothova.lubomira@gmail.com

Phone: +421 2 59357296

Fax: +421290119-631

1 Supplementary Methods

Neutrophil isolation

Neutrophils were isolated from the bone marrow of wildtype C57/Bl6J and PAD4−/− mice according to previously published protocol [1]. Mice were euthanized, femurs and tibias were flushed with RPMI + 1% FCS and cells were passed through a 70 μm strainer to remove tissue. Red blood cells were hypotonically lysed using 0.2% NaCl, followed by the addition of 1.2% NaCl to stop lysing. Neutrophils were then isolated by density gradient centrifugation percoll (GE Healthcare, Chicago, IL, USA). Cells were gently laid on percoll and centrifuged at 550g for 35 min at RT without breaks. Neutrophils were then collected and used for subsequent experiments.

Fluorescence microscopy

Detection of NETs formation in murine neutrophils via fluorescence microscopy was performed using a modified method published earlier [2]. Neutrophils at a concentration of 1^ 10^6 cells/ml were seeded on coverglasses in RPMI 1640 without phenol red (PAN-Biotech, Aidenbach, Germany), supplemented with 10% FCS (PAN-Biotech, Aidenbach, Germany), waited for adhesion for 30 min (37°C, 5% CO2) and then stimulated with uropathogenic *E. Coli* (2^10^7 cells/ml) or 500 nM phorbol 12-myristate 13-acetate (Sigma-Aldrich, St. Louis, MO, USA) for 3 hours. Cells were then fixed with 2% paraformaldehyde, blocked with 5% bovine serum albumin in PBS and permeabilized by 0.05% Triton-X100. Coverslips were then stained with primary Anti-Histone H3 (citrulline R2 + R8 + R17) antibody (ab5103) (Abcam, Cambridge, UK) at 1 μg/ml, followed by secondary Alexa Fluor® 555

Donkey anti-rabbit IgG (minimal x-reactivity) antibody (Biolegend, San Diego, CA, USA) at 2 µg/ml. DNA was stained with 200 nM SYTOX GreenTM (Thermo Fisher, *Waltham*, *MA*, USA). The studies were performed on a fluorescent inverted microscope Axio Lab.A1 (Zeiss, Oberkochen, Germany).

2 Supplementary Results

Leukocyturia positively correlated with urinary MPO ($r=0.29$, $p<0.05$, Supplementary figure 1A) and cathelicidin ($r=0.27$, $p<0.05$, Supplementary figure 1B). Although urinary total ecDNA and ncDNA did not correlate with leukocyturia (ecDNA: $r=0.05$, $p>0.05$; ncDNA: $r=-0.007$, $p>0.05$, data not shown) we found trend to positive correlation between urinary mtDNA and leukocyturia ($r=0.11$, $p>0.05$, Supplementary figure 1C).

3 Supplementary Figures

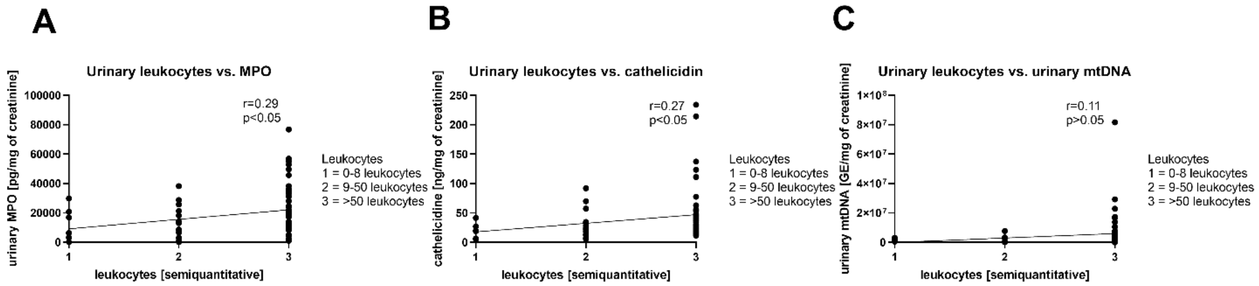

Supplementary Figure 1. Correlation between leukocyturia and markers of NETs in urine of children with urinary tract infection (UTI).

Relation between (**A**): myeloperoxidase (MPO), (**B**): cathelicidin, (**C**): mitochondrial (mtDNA) and leukocyturia. Relations were tested using Spearman´s rank correlation test. P values less than 0.05 are considered as statistically significant.

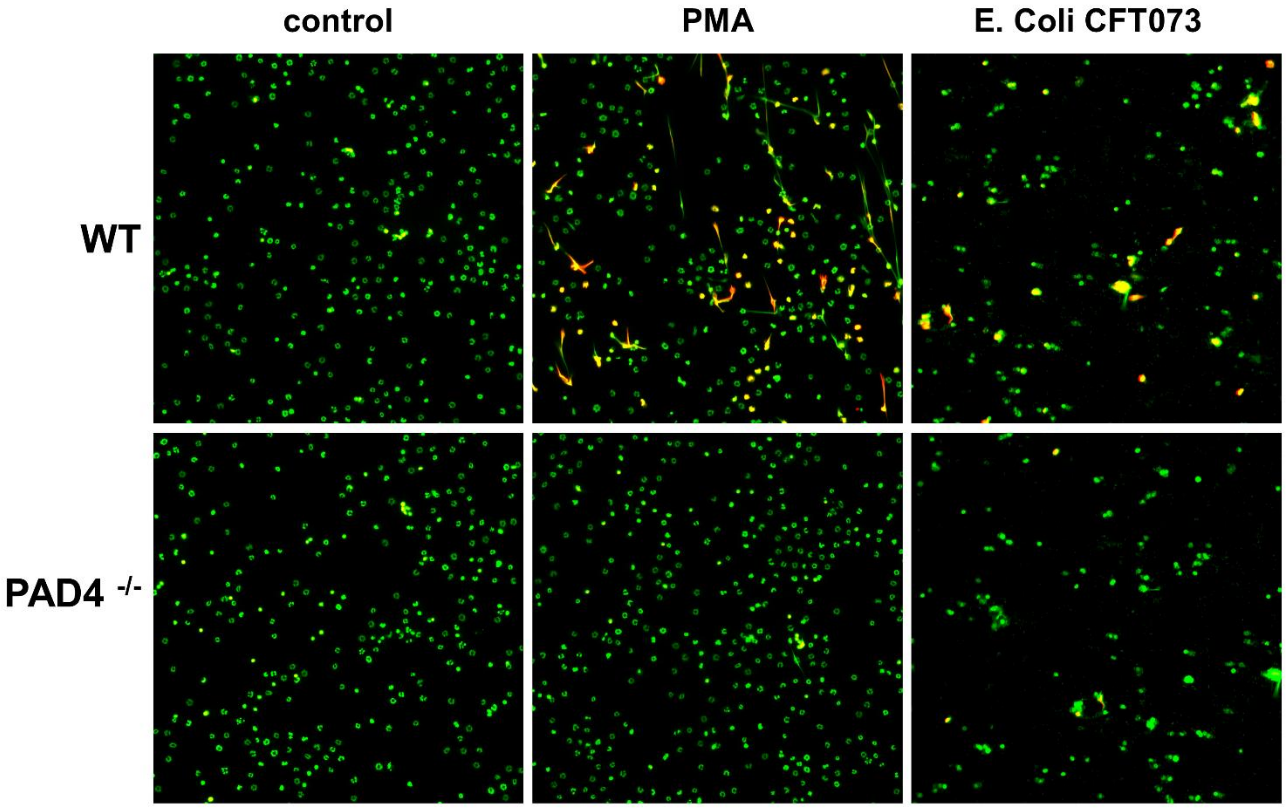

Supplementary figure 2: Representative images of NETs formation induced in polymorphonuclear cells (PMNs) of WT and PAD4-/- mice observed by fluorescence microscopy (40x magnification). PMNs isolated from bone marrow of both WT (n=3) and PAD4-/- (n=3) mice were incubated in the presence of 500 nM phorbol 12-myristate 13-acetate (PMA) and uropathogenic *E. Coli* (MOI 20) for 4 hours, fixed and stained with DNA-binding dye SYTOX GreenTM (green channel) together with antibody against citrullinated histone H3 (red channel). Colocalization of signal from green channel with strong signal from red channel in nuclei with altered morphology indicates the presence of NETs.

4 Supplementary references:

1. Swamydas M, Luo Y, Dorf ME, Lionakis MS. Isolation of Mouse Neutrophils. Curr Protoc Immunol. 2015;110: 3.20.1-3.20.15. doi:10.1002/0471142735.im0320s110

2. Brinkmann V, Laube B, Abu Abed U, Goosmann C, Zychlinsky A. Neutrophil Extracellular Traps: How to Generate and Visualize Them. J Vis Exp. 2010. doi:10.3791/1724
